# Impulse oscillometry and its independent role in the diagnosis of chronic obstructive pulmonary disease

**DOI:** 10.1016/j.heliyon.2023.e23627

**Published:** 2023-12-13

**Authors:** Kuang-Yu Chen, Ming-Hui Hung, Kuo-Chin Kao

**Affiliations:** a Chest Medicine, Lo-Tung Poh-Ai Hospital, No. 83, Nanchang Street, Luodong Township, Yilan County 265, Taiwan; b Chang Gung University College of Medicine, Taoyuan, Taiwan; c Department of Thoracic Medicine, Chang Gung Memorial Hospital, Linkou, Taiwan

**Keywords:** Chronic obstructive pulmonary disease, Impulse oscillometry, Spirometry, Body mass index

## Abstract

**Background:**

Pulmonary function test, particularly in patients with COVID-19, is problematic because it involves forced expiration. Impulse oscillometry (IOS) reduces the potential exposure of health-care staff to infectious droplets. In this study, we investigated the correlation between IOS and spirometry and whether IOS can precisely predict spirometry-based diagnoses of chronic obstructive pulmonary disease (COPD).

**Methods:**

We retrospectively analyzed the data (January 1 to December 31, 2021) of patients who underwent both spirometry and IOS on the same date. One-way analysis of variance was performed to evaluate the IOS results of patients stratified into two (COPD and non-COPD) groups by spirometry results. IOS results were also analyzed using receiver operator characteristics curves to diagnose advanced COPD, which was indicated by a postbronchodilator (BD) forced expiratory volume in 1 s (FEV1)/forced vital capacity (FVC) ratio of <0.6. We further evaluated the accuracy of oscillometry as a predictor of spirometry-based COPD diagnosis.

**Results:**

A total of 115 patients were included in the analysis. The best parameters assessed for spirometry-based COPD diagnosis were area under reactance (AX) and airway resistance (predicted R5% × resonant frequency) in relation to body mass index (BMI). However, when the post-BD FEV1/FVC ratio was <0.6, BMI-adjusted airway resistance had an area under curve (0.782; 95 % confidence interval: 0.620–0.945) value larger than the corresponding AX. A BMI-adjusted airway resistance value of >160 moderately predicted spirometry-based COPD diagnosis.

**Conclusions:**

BMI-adjusted airway resistance is a potential predictor of spirometry-based COPD diagnosis; the cutoff values of this parameter differ between individuals with and without obesity.

## 1 Introduction

Spirometry is essential for diagnosing pulmonary diseases. However, the levels of cooperation and effort required from patients for executing expiratory maneuvers are higher in spirometry than in other assessment methods; this leads to underdiagnosis and variations in test result quality [1]. Moreover, only 30 % of all patients can perform expiratory maneuvers that satisfy the quality standards set by the European Respiratory Society and American Thoracic Society, and this proportion is even lower in older patients [2].

After 2004, impulse oscillometry (IOS) emerged as the simplest method for calculating airway resistance and reactance [3]. In clinical practice, when predicted FEV1% is >80 %, other cutoff values may be used to diagnose the small airway phenotype of asthma: <60 % for maximal mid-expiratory flow (predicated MMEF%), >150 % for predicted R5% (R5%pred), and >0.10 kPa/L/s for R5–R20 [4]. Another set of suggested cutoff values are as follows: 0.30 kPa/L/s for R5; 0.015 kPa/L/s for R5–R20; 0.30 kPa/L/s for area under reactance (AX); and 11.23 Hz for resonant frequency (Fres). Fres has the largest area under curve (AUC) value of 0.665 (*p* = 0.001) [5].

Most prominent age-dependent changes are noted in respiratory reactance (X5), AX, Fres, and R5–R20 [6]. Annual changes in R5, X5, and Fres are greater in patients with acute exacerbation of chronic obstructive pulmonary disease (COPD) than in those without this condition [7]. The long-term variability in R5 (*p* < 0.05) may be associated with the severity of COPD, as classified using the Global Initiative for Chronic Obstructive Lung Disease [8]. A reduction in X5 may indicate emphysema [9]. IOS parameters can help diagnose COPD in chronic smokers a cutoff AX of ≥8.66 cmH_2_O/L is associated with the highest AUC (0.79), with a sensitivity of 79.1 % and a specificity of 78.0 % for COPD diagnosis [10].

According to the World Health Organization, body mass index (BMI) of 30 kg/m^2^ indicates obesity; this value is set at >27.5 kg/m^2^ in Asian individuals [11]. Body weight and BMI decrease with decreasing COPD severity. High BMI values interfere with IOS parameters, but not spirometry parameters [12]. The degree of obesity is strongly correlated with increased peripheral resistance and reduced reactance in IOS [13]. COPD overdiagnosis is more common in individuals with higher BMI [14]. It is also a SAD phenotype with reduced emphysema, preserved lung diffusion, and slow lung function decline [15].

For individuals experiencing a rapid decline in lung function, a low FEV1/forced vital capacity (FVC) of <0.6 may indicate advanced COPD. Drummond et al. analyzed data from the Lung Health Study cohort and reported that “individuals with lower baseline FEV1/FVC ratio have more rapid decline and worse mortality” [16]. The threshold FEV1/FVC and Z-scores for identifying individuals at an increased risk of a rapid decline in FEV1 should be set to lower than 0.65 and −2.0, respectively [17].

IOS exhibits high sensitivity for SAD diagnosis. For patients with COPD with FEV1% of <50 %, the best IOS parameters are the combined absolute value of X5 and (R5–R20)%R5 or that of X5, Fres, AX, and R5–R20 [18]. COPD specialists suggest that the ratio of post-bronchodilator (BD) FEV1/FVC should be < 0.7; a repeat spirometry assessment is necessary when the ratio is 0.65–0.75 [19].

IOS has been widely used worldwide to assess pulmonary function. The use of IOS as a tool for diagnosis confirmation has attracted attention in Taiwan [20]. IOS reduces the potential exposure of health-care professionals to infectious droplets. Forced expiration can lead to the production of oral droplets of varying sizes; these droplets may contain infectious particles. “While large droplets fall quickly to the ground, small droplets can dehydrate and linger as bioaerosols in the air, thus functioning as vectors for viral transmission.” Therefore, forced spirometry may pose a threat to health-care professionals because of the potential generation of infectious bioaerosols [21,22].

In this primary clinical study, we introduced a novel formula for predicting FEV1/FVC ratios of <0.7. Given that the spirometry-based cutoff for COPD diagnosis is determined on the basis of BMI, R5%, and Fres, we analyzed these three parameters in our study. We investigated the ability of BMI-adjusted airway resistance to predict spirometry-based diagnosis of COPD. BMI-adjusted airway resistance was calculated through IOS by using the following formula: (R5%pred × Fres)/BMI. The primary rationale for employing a formula rather than the traditional practice of interpreting multiple IOS items was to streamline the application of IOS in practical postpandemic situations. Our approach helps limit unnecessary forced expiration during IOS, thereby preventing health-care professionals from potentially being exposed to infected oral droplets.

## 2 Patients and methods

### 2.1 Ethics

This retrospective study was approved by the Institutional Review Board (IRB) of Hualien Tzu Chi Hospital, Buddhist Tzu Chi Medical Foundation (approval number: IRB111-147-B; approval date: July 14, 2022). The requirement for informed consent was waived by the IRB.

### 2.2 Patients

Data were collected for the period between January 1 and December 31, 2021. During the outbreak of COVID-19 in Taiwan from 2021 to 2022, spirometry and IOS were completed separately in our hospital on the same day or within a 4-week interval to ensure compliance with the hitherto pandemic control measures. Initially, 325 patients were included; of them, 115 were included in the final analysis. Spirometry was performed for asthma, bronchiectasis, and COPD. Patients without spirometry data (n = 197) and those with incomplete IOS data (n = 13) were excluded from this study. Bronchodilation tests were performed in patients whose FVC and FEV1 were <80 % of the predicted values. Patients’ tobacco use (smoking) status was recorded.

### 2.3 Measurements and Statistical analysis

Statistically proportional quintile classification was performed using the post-BD FEV1/FVC ratio. The first, second, and third quintiles represented the lowest, moderate, and highest (20 %) values, respectively. Data corresponding to the first quintile were used for diagnosing advanced COPD on the basis of a post-BD FEV1/FVC ratio of <0.6, which is indicative of a rapid decline in lung function [16,17]. The aforementioned definitions were obtained from studies on small airway dysfunction [23] and COPD [24].

BMI-adjusted airway resistance was measured using the following formula: (R5%pred × Fres)/BMI. This parameter indicated the efficacies and roles of individual parameters in predicting spirometry-based COPD diagnosis. Receiver operating characteristic (ROC) curves were used to evaluate the diagnostic efficacies and reference values of different IOS and spirometry parameters. Correlation analysis was performed using Spearman's test. Statistical significance was set at *p* < 0 0.05.

Continuous data exhibiting a normal distribution are presented as mean ± standard deviation values, and those exhibiting a nonnormal distribution are presented as median values. Categorical variables (e.g., number of patients) were compared using the chi-squared test, whereas continuous variables were compared using the *t*-test. Pearson correlation analysis was performed to determine pairwise correlations between continuous variables, and multivariable linear regression was performed to identify the independent factors influencing the dependent variable (post-BD FEV1/FVC ratio). The following variables were included in univariate analysis: age, sex (female/male), BMI, smoking habit (non/former/current), packs/year, post-BD FEV1 and FEV1%, post-BD FEV1/FVC ratio, and pre- and post-BD MMEF ratios. Variables with significant results (*p* < 0.1) were included in a subsequent stepwise multivariable regression model. ROC curves were plotted to present the true positive rate (sensitivity) as a function of the false positive rate (specificity). All analyses were performed using SPSS (version 22.0; IBM Corporation; Armonk, NY, USA).

## 3 Theory

Our findings indicate the potential roles of three parameters (resistance, reactance, and BMI) in the prediction of COPD for chest physicians and clinicians.

## 4 Results

### 4.1 Baseline characteristics

A total of 115 patients (men, 61 [53.0 %]; mean age, 62.9 ± 15.7 years) were included in the final analysis. Of the patients, 18 (15.7 %) were current smokers, and 27 were ex-smokers. Most patients had clinically diagnosed asthma (44.3 %), followed by hypertension and hypertensive cardiac disease (39.1 %) and allergic rhinitis (31.3 %). Other comorbidities, such as gastroesophageal reflux disorder (21.7 %), diabetes mellitus (20.0 %), and coronary artery disorder (10.4 %), were also noted; however, these comorbidities did not exhibit acute deterioration during the study period. Bronchiectasis was diagnosed in 8 patients (7 %), but no patient received a diagnosis of interstitial lung disease. A total of 56 patients underwent bronchodilation tests, and 25 (21.7 %) were responsive to BD.

Of the patients, 30 (26.1 %) satisfied the spirometry-based criteria for COPD diagnosis; of them, 10 were responsive to BD. Significant differences were observed in tobacco-smoking years between patients with COPD and those without COPD (24.0 ± 24.8 and 11.2 ± 20.2 pack-years, respectively; *p* < 0.01). Among the 30 patients who met the spirometry-based diagnostic criteria for COPD, 12 were never-smokers, 4 were current smokers, and 14 were ex-smokers. Furthermore, 13 of the 30 patients had a clinical history of asthma. For the spirometry-based diagnosis of COPD in a real-world setting, the personal history of each patient was recorded; notably, personal history or any indication of a comorbidity did not preclude the potential coexistence of COPD and other conditions. All 115 patients underwent complete spirometry with pre-BD tests. For a spirometry-based confirmation of COPD diagnosis, the post-BD FEV1/FVC ratio should be < 0.7. The ratio for 56 patients met this criterion. The pre-BD FEV1/FVC ratio was >0.7 for the remaining 59 patients; therefore, further BD tests were considered unnecessary for these patients. This is a standard practice in real-world spirometry interpretation by pulmonary physicians.

A total of 34 (29.6 %) patients had obesity (BMI >28 kg/m^2^); no considerable age difference was noted between these patients and those without obesity (n = 81). The patients with obesity had significantly higher R5%pred and R20 values than did those without obesity (*p <* 0.05). A positive correlation was observed between BMI and R5%pred (0.274; *p* < 0.05). Significant between-sex differences were observed in AX (*p <* 0.01). This real-world observational study included outpatients with COPD and other diseases. Table 1 presents patient characteristics stratified by BMI and spirometry-defined COPD. This table also shows the differences in post-BD FEV1/FVC ratios between individuals with a BMI of ≥28 kg/m^2^ and those with a BMI of <28 kg/m^2^ and between individuals with COPD and those without COPD.

**Table 1:** Characteristics of patients stratified by body mass index and by spirometry-defined COPD.

	BMI ≧ 28 (n = 34)		BMI <28 (n = 81)		*p*-value	COPD (n = 30)		non-COPD (n = 85)		*p*-value
Variables	Mean (N)	SD (%)	Mean (N)	SD (%)		Mean (N)	SD (%)	Mean (N)	SD (%)	
Age (years)	61.9	15.6	63.3	15.9	*0.657*	67.6	13.8	61.3	16.1	*0.058*
Body weight	80.8	13.8	61.3	11.0	*<0.001*	66.3	14.6	67.4	15.0	*0.726*
Body height	158.7	8.9	160.9	9.1	*0.239*	160.9	9.8	160.1	8.9	*0.683*
BMI	32.1	5.2	23.6	3.1	*<0.001*	25.7	6.1	26.2	5.3	*0.667*
Asthma (J45)	19	16.7 %	32	28.1 %	*0.119*	13	11.4 %	38	33.3 %	*0.859*
Hypertension (I10 and I11)	18	15.7 %	27	23.5 %	*0.049*	13	11.3 %	32	27.8 %	*0.587*
Rhinitis (J30 and J3x)	8	7.2 %	28	25.2 %	*0.183*	9	8.1 %	27	24.3 %	*0.853*
Gastroesophageal reflux disorder (K21 and K2x)	6	5.2 %	19	16.5 %	*0.491*	5	4.3 %	20	17.4 %	*0.438*
post-BD FEV1/FVC ratio*	0.76	0.08	0.74	0.12	*0.475*	0.60	0.10	0.80	0.05	*<0.001*
post-BD FVC	2.50	0.73	2.66	0.98	*0.372*	2.39	0.82	2.69	0.93	*0.121*
post-BD FVC%pred	0.90	0.23	0.88	0.20	*0.768*	0.85	0.27	0.90	0.18	*0.316*
post-BD FEV1	1.91	0.74	1.98	0.87	*0.687*	1.47	0.62	2.14	0.83	*<0.001*
post-BD FEV1%pred	0.85	0.24	0.82	0.21	*0.499*	0.66	0.26	0.89	0.17	*<0.001*
pre-BD MMEF%pred	0.69	0.35	0.64	0.29	*0.422*	0.31	0.15	0.77	0.26	*<0.001*
post-BD MMEF%pred**	0.62	0.28	0.52	0.29	*0.239*	0.35	0.17	0.74	0.24	*<0.001*
R5	0.56	0.21	0.47	0.21	*0.040*	0.56	0.27	0.47	0.18	*0.047*
R5%pred	160.03	55.99	133.76	52.51	*0.018*	167.93	74.41	132.21	42.49	*0.002*
R20	0.36	0.09	0.32	0.11	*0.044*	0.33	0.09	0.33	0.11	*0.889*
R5–R20	0.19	0.15	0.15	0.15	*0.126*	0.23	0.22	0.14	0.11	*0.004*
X5	−0.19	0.17	−0.20	0.16	*0.744*	−0.31	0.25	−0.16	0.10	*<0.001*
Fres	18.65	4.46	18.62	6.31	*0.978*	21.26	6.26	17.70	5.38	*0.003*
AX	1.63	1.52	1.58	1.75	*0.871*	2.61	2.43	1.23	1.14	*<0.001*

*: all the items in %pred are presented with original%pred*100.**: post-BD MMEF case numbers equaled to 56 and 17 cases were in obese, 39 cases were in non-obese; The units of IOS variables R5, R20, R5-R2, X5 are kPa/L/s; Fres: Hz, AX: kPa/L. The units of spirometry variables FVC, FEV1, MMEF are liter/sec; GERD: gastroesophageal reflux disorder. Values in each of mean and SD of asthma, hypertension, rhinitis and GERD represented the cases numbers and percentage in the whole study cases, and *p*-values of these comorbidites represents the Pearson's chi-square test.

In this table, the independent samples *t*-test comparing the spirometry and the IOS means of two independent groups (COPD and non-COPD) revealed there are statistical evidence that the associated population means are significantly different on post-BD FEV1/FVC ratio, post-BD FEV1, post-BD FEV1%pred, pre- and post-BD MMEF%pred, X5 and AX (all *p <* 0.001). Levene's test for equality of variances suggested that the variances of the homogeneity of variances assumption in COPD and non-COPD is violated on post-BD FVC, post-BD FVC%pred, post-BD FEV1, pre- and post-BD MMEF%pred, R5, R20 and Fres.

The data presented in this table clearly explain the rationale for introducing our formula: [(R5%pred × Fres)/BMI]. R5, R5%pred, and R20 varied significantly between the patients with a BMI of ≥28 kg/m^2^ and those with a BMI of <28 kg/m^2^ (*p* < 0.05). However, these two cohorts did not differ significantly in terms of AX (Table 1). Our findings indicate that proximal and whole airway resistance values are higher in patients with obesity than in those without it (BMI <28 kg/m^2^).

Pearson correlation coefficients were 0.746 for R5 and R20 and −0.413 for R5–R20 in pre-BD MMEF and −0.421 for body height and AX (all *p* < 0.05).

**Fig. 1:**
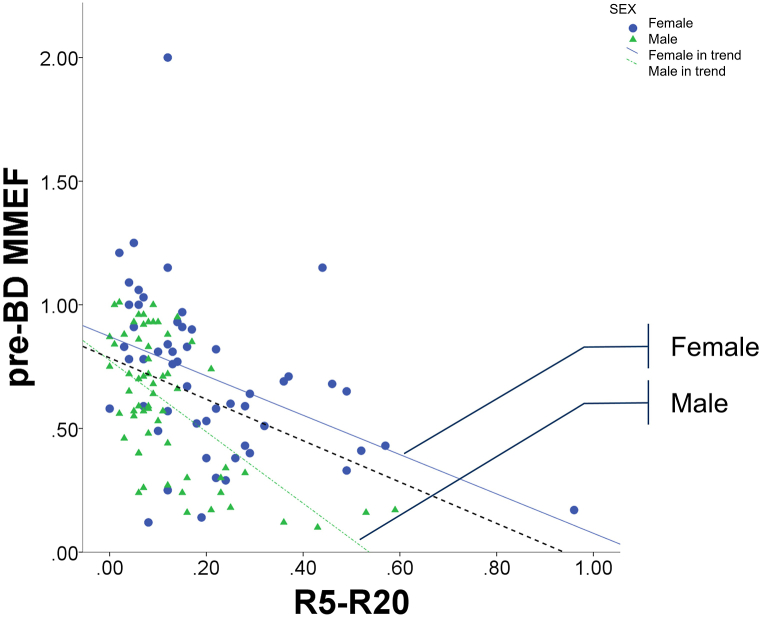
Correlation between pre-BD predicted MMEF% and R5–R20 in all patients. The black dots represent female patients, white triangles represent male patients, black dashed line represents the trend line of the correlation between pre-BD MMEF and R5–R20 for all patients, black solid line represents the trend line of the aforementioned correlation for female patients, and gray dotted line represents the trendline of the aforementioned correlation for male patients. The units of the pre-BD MMEF and R5–R20 are decimal and kPa/L/s, respectively. BD, bronchodilator; MMEF, maximal mid-expiratory flow; R5–R20, difference in airway resistance measured at 5 and 20 Hz in forced oscillation.

The median R5 and R20 values were 0.54 ± 0.23 and 0.37 ± 0.12 kPa/L/s, respectively in women. They were 0.38 ± 0.16 and 0.29 ± 0.07 kPa/L/s respectively in men (*p* < 0.001). The median R5–R20 and pre-BD MMEF values are presented in Fig. 1. R5–R20 was significantly higher (*p* < 0.05) in women than in men and were significantly correlated with AX (0.951; *p* < 0.001).

### 4.2 Differences between COPD and other airway diseases

R5%pred was higher in patients with COPD than in those without COPD (167.9 ± 74.4 % and 132.2 ± 42.5 %, respectively; *p < 0.01*). R5% pred, Fres, and AX could moderately and individually predict COPD.

Table 2 (row numbers 1 and 2) presents the results of spirometry-based COPD or advanced COPD prediction by different IOS items and BMI-adjusted airway resistance. The table also includes the different cutoff values of BMI-adjusted airway resistance for predicting the spirometry results presented in rows 3 (BMI <28 kg/m^2^) and 4 (BMI ≥28 kg/m^2^).

**Fig. 2:**
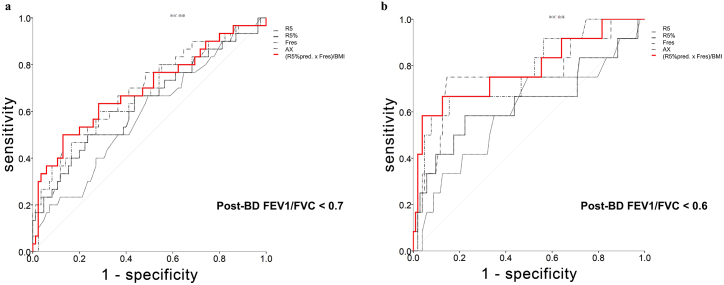
Area under curves (AUC) corresponding to the parameters assessed for the spirometry-based diagnosis of (A) chronic obstructive pulmonary disease (COPD; a postbronchodilator forced expiratory volume in 1 s [FEV1]/forced vital capacity [FVC] ratio of <0.7) and (B) advanced COPD (a postbronchodilator FEV1/FVC ratio of <0.6). The following five parameters are indicated: resistance at 5 Hz (R5; thin black line), predicted R5% (R5%pred; thick black line), resonant frequency (Fres; thin dashed line), area under reactance (AX; thin gray line), body mass index (BMI)–adjusted airway resistance ([R5%pred × Fres]/BMI; red line). (For interpretation of the references to colour in this figure legend, the reader is referred to the Web version of this article.)

BMI-adjusted airway resistance had the best AUC value (0.70 ± 0.06; 95 % CI: 0.58–0.82; Table 2; Fig. 2A).

**Table 2:** Area under curve values corresponding to the assessed parameters.

Test parameters	Area	Standard error	Asymptotic significance^b^	Asymptotic 95 % confidence interval		Sensitivity	Specificity
				Lower limit	Upper limit		
1. Spirometry-based COPD diagnosis based on a post-BD FEV1/FVC ratio of <0.7							
R5	0.586	0.061	0.164	0.466	0.705	0.667	0.506
R5%pred	0.636	0.063	0.027	0.514	0.759	0.600	0.588
Fres	0.673	0.060	0.005	0.555	0.791	0.633	0.588
AX	0.697	0.058	0.001	0.582	0.811	0.733	0.518
BMI-adjusted	0.696	0.060	0.001	0.578	0.815	0.667	0.624
2. Spirometry-based COPD diagnosis; based on BMI-adjusted airway resistance							
100	0.628	0.059	0.037	0.512	0.745	0.633	0.624
110	0.637	0.061	0.026	0.518	0.757	0.533	0.741
150	0.636	0.064	0.027	0.512	0.761	0.367	0.906
160	0.654	0.064	0.012	0.529	0.779	0.367	0.941
170	0.643	0.064	0.020	0.517	0.769	0.333	0.953
3. Spirometry-based COPD diagnosis based on BMI-adjusted airway resistance in patients with a BMI value of ≥28 kg/m^2^							
100	0.690	0.113	0.125	0.468	0.913	0.714	0.667
110	0.675	0.122	0.160	0.436	0.913	0.571	0.778
150	0.606	0.131	0.394	0.349	0.862	0.286	0.926
160	0.624	0.132	0.317	0.365	0.883	0.286	0.963
170	0.553	0.130	0.670	0.299	0.807	0.143	0.963

1, Diagnosis based on R5, R5%pred, R5–R20, Fres, and AX; at least one common factor between the positive and negative actual state groups.

2 and 3, Diagnosis based on different cutoff values of BMI-adjusted airway resistance: 100, 110, 150, 160, and 170; at least one common factor between the positive and negative actual state groups.

AX, area under reactance; BMI, body mass index; COPD, chronic obstructive pulmonary disease; Fres, resonant frequency; R5, resistance at 5 Hz; and R5%pred, predicted R5%.

Statistics may be biased.

^a^Nonparametric assumption.

bNull hypothesis: true area, 0.5.

### 4.3 Spirometry-based advanced COPD diagnosis

Advanced obstruction in spirometry was analyzed by comparing IOS parameters. Fres was the best individual parameter for predicting advanced COPD (AUC: 0.77 ± 0.08; 95 % CI: 0.62–0.92), followed by AX (AUC: 0.76 ± 0.09; 95 % CI: 0.59–0.92; both *p* < 0.05; Table 2). BMI-adjusted airway resistance had the best AUC value (0.78 ± 0.08; 95 % CI: 0.62–0.95; *p <* 0.01; Fig. 2B).

### 4.4 Multivariable linear regression

The F-ratios obtained through the analysis of variance performed using Model 3 (Table 3) indicated that the independent variables (BMI-adjusted airway resistance, R5–R20, and AX) significantly predicted the dependent variable (F = 16.363; *p* < 0.01; R^2^ = 0.554). Two variables (R5–R20 and AX) contributed to significance of the prediction (*p* < 0.05). The Dubin–Watson statistical value of Model 3 was 1.973, which indicated a considerably weak, positive autocorrelation between these variables. However, the variance inflation factor value corresponding to BMI-adjusted airway resistance was 1.947 (Table 4). This was the only variable with a value of <10; therefore, the collinearity of BMI-adjusted airway resistance was the least significant. In summary, Model 3 provided the best prediction of the dependent variable.

**Table 3:** Multivariable linear regression model for postbronchodilator FEV1/FVC.

Model^b^	R^a^	R square	Adjusted R^2^	Standard error	Changes					Durbin–Watson statistics
					Changes in R^2^ values	Changes in F values	df1	df2	Significant changes in F values	
1	0.653	0.426	0.389	0.0857	0.426	11.361	7	107	0	2.072
2	0.551	0.303	0.271	0.09357	0.303	9.491	5	109	0	1.971
3	0.554	0.307	0.288	0.09250	0.307	16.363	3	111	0	1.973

AX, area under reactance; BH, body height; BMI, body mass index; BW, body weight; Fres, resonant frequency; R5, resistance at 5 Hz; R5%pred, predicted R5%; and R5–R20, difference in airway resistance measured at 5 and 20 Hz in forced oscillation.

a Predictors, 1: constant, AX, BMI, Fres, BH, R5, R5value, and BW; 2: constant, AX, BMI, Fres, R5%pred, and R5–R20; and 3: constant, BMI-adjusted airway resistance, R5–R20, and AX.

b Dependent variable: postbronchodilator forced expiratory volume in 1 s (FEV1)/forced vital capacity (FVC).

**Table 4:** Model coefficients and statistical significance.

Model^a^	Variables	Unstandardized coefficients		Standardized coefficients	T	Significance	95 % confidence interval		Correlation			Collinearity	
		Beta	Standard error	Beta			Lower limit	Upper limit	Zero-order	Partial	Part	Tolerance	Variance inflation factor
1	Constant	0.464	0.689		0.673	0.502	−0.903	1.831					
	BW	−0.002	0.005	−0.319	−0.518	0.606	−0.011	0.007	0.059	−0.05	−0.038	0.014	70.837
	BH	0.002	0.004	0.152	0.427	0.670	−0.007	0.010	−0.037	0.041	0.031	0.042	23.6
	BMI	0.006	0.011	0.308	0.537	0.593	−0.017	0.029	0.080	0.052	0.039	0.016	61.391
	R5	0.691	0.152	1.351	4.531	0.000	0.388	0.993	−0.210	0.401	0.332	0.060	16.585
	R5%pred	−0.002	0.000	−0.812	−3.287	0.001	−0.003	−0.001	−0.317	−0.303	−0.241	0.088	11.375
	Fres	−0.001	0.002	−0.070	−0.801	0.425	−0.005	0.002	−0.352	−0.077	−0.059	0.705	1.419
	AX	−0.061	0.010	−0.942	−5.887	0.000	−0.082	−0.041	−0.453	−0.495	−0.431	0.209	4.779
2	Constant	0.831	0.056		14.71	0.000	0.719	0.943					
	BMI	0.000	0.002	−0.023	−0.252	0.802	−0.004	0.003	0.080	−0.024	−0.020	0.790	1.266
	R5%pred	−5.69E-05	0.000	−0.028	−0.195	0.845	−0.001	0.001	−0.317	−0.019	−0.016	0.304	3.295
	R5–R20	0.654	0.219	0.909	2.993	0.003	0.221	1.088	−0.340	0.276	0.239	0.069	14.417
	Fres	−0.002	0.002	−0.106	−1.148	0.254	−0.005	0.001	−0.352	−0.109	−0.092	0.746	1.34
3	Constant	0.796	0.017		45.75	0.000	0.762	0.831					
	R5–R20	0.611	0.193	0.848	3.163	0.002	0.228	0.994	−0.340	0.288	0.250	0.087	11.516
	AX	−0.075	0.019	−1.157	−3.905	0.000	−0.114	−0.037	−0.453	−0.348	−0.309	0.071	14.043
	BMI-adjusted airway resistance	0.000	0.000	−0.157	−1.425	0.157	−0.001	0.000	−0.447	−0.134	−0.113	0.514	1.947

AX, area under reactance; BH, body height; BMI, body mass index; BW, body weight; Fres, resonant frequency; R5, resistance at 5 Hz; R5%pred, predicted R5%; and R5–R20, difference in airway resistance measured at 5 and 20 Hz in forced oscillation.

a Dependent variable: postbronchodilator forced expiratory volume in 1 s/forced vital capacity.

## 5 Discussion

### 5.1 Principal findings

In this retrospective study in northeastern Taiwan, we investigated the correlation between spirometry and IOS in patients with clinical upper or lower respiratory symptoms. To the best of our knowledge, the formula (R5%pred × Fres)/BMI has never been used for the IOS-based prediction of spirometry-based COPD diagnosis. Several IOS parameters have been explored previously; however, in most studies, only one parameter or two parameters have been evaluated. In the present study, we specifically included patients with various pulmonary disorders who reported to our pulmonary care outpatient department with lower respiratory tract symptoms. The demographic characteristics of our cohort are almost like those noted by pulmonary physicians or general medicine physicians in clinical practice. Although other studies including patients with COPD have reported higher AUC values for individual IOS parameters, these values appeared to be overestimation of the corresponding predictabilities in real-world clinical practice. Therefore, we developed a formula for COPD prediction that combined three key parameters: (R5% × Fres)/BMI.

The aforementioned formula, which considers multiple associated parameters and patients' BMI, can be used to predict if patients’ post-PD FEV1/FVC ratios are <0.7, which, in turn, can predict spirometry-based COPD diagnosis in patients with or without obesity. AX appears to be the best individual predictor of COPD diagnosis (AUC: 0.70 ± 0.06; 95 % CI: 0.58–0.81) when the cutoff value for prediction is 2.61 ± 2.43 kPa/L, followed by Fres (AUC: 0.67 ± 0.06; 95 % CI: 0.56–0.79), when the cutoff value is 21.26 ± 6.26 Hz.

### 5.2 R5–R20 and BMI/post-BD FEV1

Statistical significance was observed for the associations of both R5%pred and R20 with obesity. However, obesity did not influence the IOS parameter of R5–R20. R5–R20, particularly R20, was higher in women than in men. In men, when the R5–R20 value increased to approximately 0.40, the pre-BD MMEF value rapidly decreased to <0.5. However, this negative correlation between R5–R20 and pre-BD MMEF was less pronounced in women; pre-BD MMEF was approximately 0.5 when R5–R20 was >0.40. This may contribute to SAD overdiagnosis in women. A significantly negative correlation was noted between R5–R20 and pre-BD MMEF. The decrease in the slope between the two parameters was slow in women. Women had significantly higher R20 values than did men. Although R5 and R20 are linearly correlated, at the same R5 value, R20 was significantly higher in women than in men. This may explain the higher R5–R20 values in women than in men.

### 5.3 BMI-adjusted airway resistance and its cutoff values for COPD prediction

An evaluation of real-world COPD cases indicated consistency between spirometry and IOS; however, IOS exhibited moderate sensitivity and specificity [18]. We found AX to be the best individual parameter for COPD prediction; it had the highest AUC value of 0.697 (95 % CI: 0.582–0.811).

**Fig. 3:**
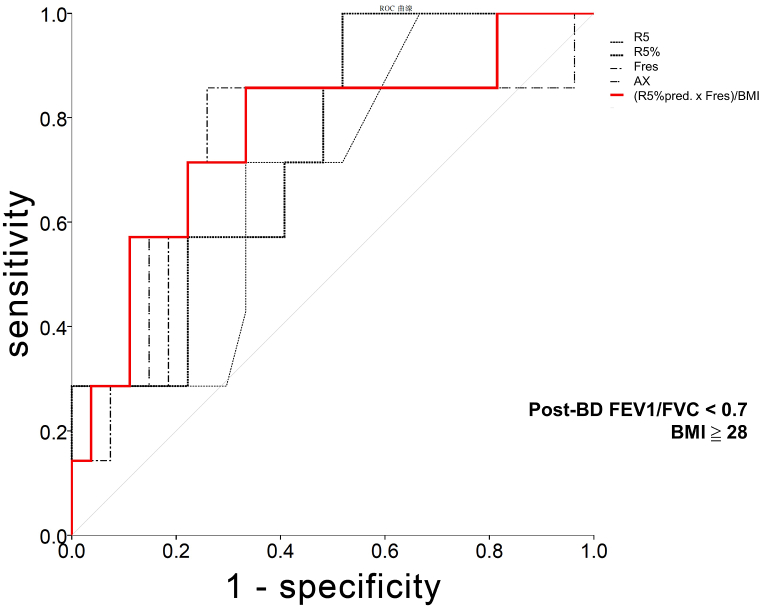
Area under curves (AUCs) corresponding to parameters assessed for the spirometry-based diagnosis of chronic obstructive pulmonary disease (a postbronchodilator forced expiratory volume in 1 s/forced vital capacity ratio of <0.7) in patients with a body mass index (BMI) of >28 kg/m^2^. The following five parameters are indicated: resistance at 5 Hz (R5; thin black line), predicted R5% (R5%pred; thick black line), resonant frequency (Fres; thin dashed line), area under reactance (AX; thin gray line), and BMI-adjusted airway resistance ([R5%pred × Fres]/BMI; red line). (For interpretation of the references to colour in this figure legend, the reader is referred to the Web version of this article.)

A BMI value of >28 kg/m^2^ was negatively correlated with the degree of airway resistance, as assessed through IOS. The AUC value corresponding to BMI-adjusted airway resistance was 0.767 (95 % CI: 0.554–0.980) for a cutoff value of 100 in patients with obesity, which indicated COPD diagnosis (*p* < 0.05; Fig. 3).

Given the current diagnostic criteria for COPD, IOS parameters appear to have high predictive value. Although AX is the first choice for COPD prediction, its AUC was only 0.70. Fres and AX showed high sensitivity and accuracy for advanced COPD diagnosis (a post-BD FEV1/FVC ratio of <0.6). The AUC of Fres was 0.77 ± 0.08 (95 % CI: 0.62–0.92) at 24.11 ± 6.13 Hz, whereas that of AX was 0.76 ± 0.09 (95 % CI: 0.59–0.92) at 3.07 ± 2.09 kPa/L. As mentioned earlier in the text, the AUC of BMI-adjusted airway resistance was the highest (0.782; 95 % CI: 0.620–0.945; Fig. 2B). Thus, we demonstrated that body weight influences both resistance and reactance. The prediction of spirometry-based COPD diagnosis in patients with various diseases is essential for early diagnosis and effective treatment. Although our formula includes only three parameters, its AUC for COPD diagnosis (an FEV1/FVC ratio of <0.7 COPD) is noninferior to that reported in another study, in which an FEV1% value of <50 % indicated COPD [18].

**Fig. 4:**
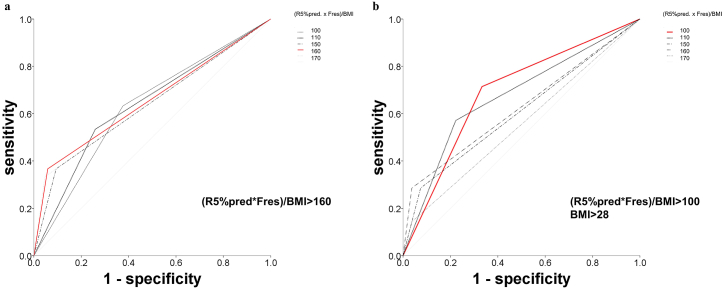
Area under curves (AUCs) corresponding to the impulse oscillometry parameters used to predict spirometry-based chronic obstructive pulmonary disease (COPD) diagnosis (a postbronchodilator forced expiratory volume in 1 s/forced vital capacity ratio of <0.7). (A) Body mass index (BMI)–adjusted airway resistance ([predicted R5% × resonant frequency])/BMI) with five cutoff values: 100 (thin black dotted line), 110 (black dotted line), 150 (dashed line), 160 (red line), and 170 (thin gray dashed line). The AUC for COPD prediction was 28, and BMI was >28 kg/m^2^ (B) BMI-adjusted airway resistance with five cutoff values: 100 (red line), 110 (black dotted line), 150 (dashed line), 160 (long gray dashed line), and 170 (thin gray dashed line). (For interpretation of the references to colour in this figure legend, the reader is referred to the Web version of this article.)

In ROC curve analysis, the AUC value for the prediction by BMI-adjusted airway resistance by using data of the lowest quintile was 0.799 (95 % CI: 0.690–0.908). Considering the median value of the aforementioned parameter, 155.5 ± 99.7 in the lowest quintile, we selected 160 as the cutoff value for the prediction of spirometry-based COPD diagnosis in patients without obesity. A BMI-adjusted airway resistance value of >160 resulted in an AUC of 0.654 (95 % CI: 0.529–0.779), which was higher than those corresponding to the other tested cutoff values of 100, 110, 150, 160, and 170 (Table 2; Fig. 4A). In patients with obesity, the cutoff value with the highest AUC was 100 (Table 2; Fig. 4B).

### 5.4 Confounders in IOS assessment

Both R4–R20 and AX and their AUC values (0.779 and 0.752, respectively) were associated with lung functional capacity in COPD and may predict low exercise tolerance [2]. Tse et al. recently reported that reactance has high sensitivity (70%–76 %) and specificity (64%–72 %) for the prediction of severe COPD [25]. In our study, Fres had the highest AUC (0.80). However, the R values were insensitive (AUC: 0.4–0.6). Isolated R values are poor indicators of disease severity [26]. Fres and frequency dependence may help differentiate between healthy individuals and patients with COPD [27]. In this real-world study, we introduced a formula that leverages the advantages of IOS in measuring both reactance and whole airway resistance. The BMI-adjusted formula was discovered to be positively correlated with peripheral reactance and effectively predicted spirometry-based COPD diagnoses. The AUC value for prediction on the basis of BMI-adjusted airway resistance when data corresponding to the lowest quintile were used was 0.799 (95 % CI: 0.690–0.908). A BMI-adjusted airway resistance value of >160 resulted in an AUC of 0.654 (95 % CI: 0.529–0.779).

### 5.5 Limitations

The present study has some limitations. First, the sample size was small because we only included patients who underwent both spirometry and IOS on the same day. We applied an observational study design. Hence, when calculating the sample size, we did not intend to compare individuals with COPD with those without it. Our objective was to evaluate the efficacy of our prediction formula. For a population of approximately 100,000 people (confidence level: 95 %) and an error margin of 5 %, the required sample size was determined to be approximately 385. However, we initially included 325 patients from our outpatient department; of them, 115 were included in the final analysis. Therefore, our study is limited because the sample size was smaller than that required for achieving statistical power. Second, because of the retrospective nature of the study, information on disease prognosis was limited. Third, the possibility of selection biases attributable to the inclusion and exclusion criteria cannot be ignored. Fourth, the reference values for IOS parameters were from studies conducted in non-Asian populations. Finally, the clinical relevance of the differences in various IOS parameters remained unclear. In future studies, the effects of the aforementioned limiting factors should be adjusted.

## 6 Conclusions

Spirometry-based COPD diagnoses may be predicted by individual IOS parameters or their combination indicating BMI-adjusted airway resistance, which is calculated as follows: (R5%pred × Fres)/BMI. Further studies conducted by pulmonary practitioners in Taiwan and East Asia are necessary to validate our findings.

## Funding source

None.

## IRB approval number

This retrospective study was approved by the Institutional Review Board (IRB) of Hualien Tzu Chi Hospital, Buddhist Tzu Chi Medical Foundation (approval number: IRB111-147-B; approved data use period: January 1 to December 31, 2021; approval date: July 14, 2022). The requirement for informed consent was waived by the IRB.

## Data availability

The data that support the findings of this study are available from the corresponding author upon reasonable request. Cite this dataset: Chen, Kuang-Yu (2023), “IOS2022_2023_original”, Mendeley Data, V1, https://doi.org/10.17632/n52swkcrx4.1.

## CRediT authorship contribution statement

**Kuang-Yu Chen:** Writing - review & editing, Writing - original draft, Software, Project administration, Methodology, Investigation, Formal analysis, Data curation, Conceptualization. **Ming-Hui Hung:** Methodology, Conceptualization. **Kuo-Chin Kao:** Methodology, Investigation, Formal analysis, Conceptualization.

## Declaration of competing interest

The authors declare that they have no known competing financial interests or personal relationships that could have appeared to influence the work reported in this article.
